# Genome Sequence of a Divergent Avian Metapneumovirus from a Monk Parakeet (*Myiopsitta monachus*)

**DOI:** 10.1128/MRA.00284-19

**Published:** 2019-04-18

**Authors:** Hanna Retallack, Susan Clubb, Joseph L. DeRisi

**Affiliations:** a Department of Biochemistry and Biophysics, University of California, San Francisco, California, USA; b Rainforest Clinic for Birds and Exotics, Loxahatchee, Florida, USA; c Chan Zuckerberg Biohub, San Francisco, California, USA; DOE Joint Genome Institute

## Abstract

Here, we report the coding-complete genome sequence of an avian metapneumovirus from a monk parakeet (Myiopsitta monachus), identified by metagenomic next-generation sequencing during an investigation into a disease outbreak in a captive parrot breeding facility. Based on divergence from known strains, this sequence represents a new subgroup of avian metapneumovirus.

## ANNOUNCEMENT

Metapneumoviruses (genus Metapneumovirus, family Pneumoviridae) cause disease in birds and mammals, including humans. Avian strains are important pathogens of commercial poultry, causing acute upper respiratory illness that is often complicated by secondary bacterial infections in chickens and turkeys (1, 2). We observed an unusual cluster of morbidity and mortality among young parrots at a captive breeding facility that could not be explained by routine diagnostics. Difficult-to-control bacterial infections and persistent cryptosporidium infections suggested immunosuppression. This prompted our investigation into underlying infectious etiologies using metagenomic next-generation sequencing.

At necropsy of an affected monk parakeet chick, the lungs, liver, and spleen were sampled and stored at −80°C. For RNA extraction, ∼50 mg of combined tissues was homogenized in 2 ml of DNA/RNA Shield (Zymo Research) using 2.8-mm ceramic beads (Omni) on a TissueLyser II instrument (Qiagen) with 5 cycles of 30 Hz for 30 sec followed by 1 min on ice. Samples were centrifuged at 16,000 × *g* for 10 min, and 250 μl of homogenized tissue supernatant was added to 750 μl of Direct-zol (Zymo Research). RNA was extracted using a Direct-zol RNA MiniPrep Plus kit (Zymo Research) with DNase treatment (Qiagen) and quantified by Nanodrop. Sequencing libraries were prepared from 100 ng of extracted RNA with 25 pg of spike-in control RNA from the External RNA Controls Consortium (ERCC) collection (Thermo Fisher Scientific), using an NEBNext Ultra II Directional RNA library prep kit for Illumina (New England Biolabs). A water sample was processed in parallel. Paired-end 150-nucleotide (nt) sequencing on an Illumina HiSeq 4000 instrument yielded 35,053,607 raw read pairs.

A representative host database was built using all genome assemblies and mitochondrial genomes under taxonomy identifier (TaxID) 9224 (Psittacidae, parrots) available in the National Center for Biotechnology Information (NCBI) database as of 7 December 2018. Host subtraction and quality control were performed as described previously (3). The remaining 1,670,686 unique nonhost read pairs (4.8% of raw) were processed using the IDseq pathogen detection pipeline v3.2 (reference, NCBI nucleotide and nonredundant protein (nt/nr) databases, as of 1 December 2018) (4), which identified metapneumovirus reads in the sample. No other viruses were detected as credible hits by the following criteria: ≥10 mapped read pairs per million nonhost read pairs (rpM) at the nucleotide level, and ≥1 rpM at the amino acid level.

**FIG 1:**
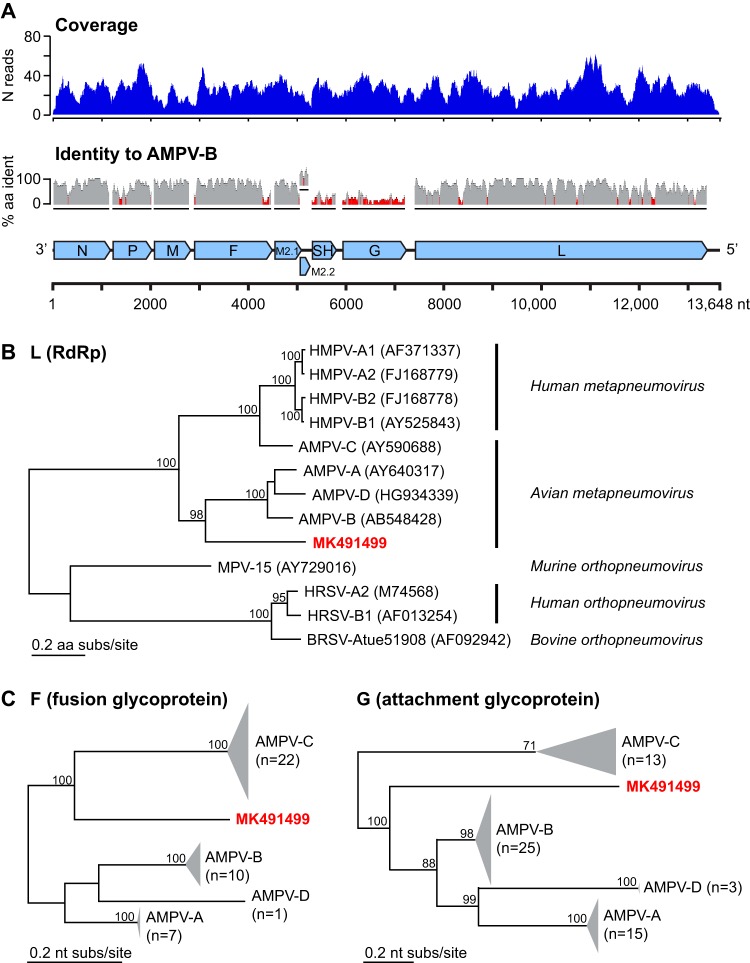
Coverage and phylogenetic analysis of sequence representing a new subgroup of avian metapneumovirus. (A) Top, coverage plot showing number of reads aligning to the consensus sequence (*y* axis) along the length of the consensus sequence (*x* axis, length in nucleotides, corresponding to the diagram of the viral genome below). Middle, percent identity (*y* axis) for a 15-amino acid sliding window across an alignment of the consensus sequence and reference AMPV-B sequence (GenBank accession number AB548428) for each viral protein. Red bars indicate an identity of <30%. Bottom, representation of likely genomic structure based on open reading frames and homology to other avian metapneumoviruses. (B) Phylogenetic tree of the *Pneumoviridae*. Amino acid level alignments of L genes (encoding RNA-dependent RNA polymerase [RdRp]) from representative viruses were used to construct a maximum likelihood tree. Multiple-sequence alignment was performed in Geneious (v9.1.8) with default parameters; the phylogenetic tree was built using PhyML v2.2.3 (LG substitution model, 100 bootstraps) (9). The sequence identified in this study is highlighted in red. Values at branch points indicate the fraction of trees with this node, based on a bootstrapping method. Bar, 0.2 amino acid substitutions per site. (C) Maximum likelihood trees (PhyML, default parameters) constructed from nucleotide alignments (Geneious, default parameters) of all available avian metapneumovirus sequences for the fusion glycoprotein (F gene, left) and attachment glycoprotein (G gene, right). Bar, 0.2 nucleotide substitutions per site.

These metapneumovirus reads were used as seeds for Paired-End Iterative Contig Extension (PRICE) v1.2 (with the settings “–fpp <R1> <R2 > 350 99 –mol 30 –target 80 8 2 2 –nc 10 –lenf 500 8”) to assemble the full-length genome (5). Reads were then mapped back to the genome using Bowtie 2 v2.2.4 (“–very-sensitive-local” mode) (6). The final consensus sequence is 13,648 nt long, with 26× mean coverage and a GC content of 39% (Fig. 1). Genome termini were not specifically identified. Consistent with active viral replication, we observed reads from both negative-strand (genomic) and positive-strand (mRNA transcript/antigenomic) RNA.

The most similar sequences in the NCBI nt/nr reference databases identified by BLAST search were metapneumoviruses (7). Phylogenetic analysis of the L gene (encoding RNA-dependent RNA polymerase [RdRp]) at the amino acid level revealed 43 to 49% identity to representative members of the genus Orthopneumovirus and 61 to 66% identity to representative members of the genus Metapneumovirus, indicating that this sequence represents a new subgroup of metapneumoviruses (Fig. 1) (8). Analysis of the fusion glycoprotein (F) gene and attachment glycoprotein (G) gene further supported this classification.

We have identified the first member of a new subgroup of metapneumoviruses, distinct from avian metapneumoviruses A, B, C, and D. Despite similarities between this outbreak and outbreaks of avian metapneumovirus in commercial poultry, it remains unknown whether the virus identified here directly caused the symptoms observed in this individual and/or flock.

### Data availability.

The avian metapneumovirus sequence described here has been deposited at GenBank under the accession number MK491499.
